# Bacterial and Fungal Microbiota Changes Distinguish *C. difficile* Infection from Other Forms of Diarrhea: Results of a Prospective Inpatient Study

**DOI:** 10.3389/fmicb.2016.00789

**Published:** 2016-05-25

**Authors:** William Sangster, John P. Hegarty, Kathleen M. Schieffer, Justin R. Wright, Jada Hackman, David R. Toole, Regina Lamendella, David B. Stewart

**Affiliations:** ^1^Division of Colon and Rectal Surgery, Department of Surgery, College of Medicine, The Pennsylvania State University, HersheyPA, USA; ^2^Department of Biology, Juniata College, HuntingdonPA, USA; ^3^Wright Labs, LLC, HuntingdonPA, USA

**Keywords:** *Clostridium difficile*, microbiome, bacterial, fungal, diarrhea

## Abstract

This study sought to characterize the bacterial and fungal microbiota changes associated with *Clostridium difficile* infection (CDI) among inpatients with diarrhea, in order to further explain the pathogenesis of this infection as well as to potentially guide new CDI therapies. Twenty-four inpatients with diarrhea were enrolled, 12 of whom had CDI. Each patient underwent stool testing for CDI prior to being treated with *difficile*-directed antibiotics, when appropriate. Clinical data was obtained from the medical record, while each stool sample underwent 16S rRNA and ITS sequencing for bacterial and fungal elements. An analysis of microbial community structures distinct to the CDI population was also performed. The results demonstrated no difference between the CDI and non-CDI cohorts with respect to any previously reported CDI risk factors. Butyrogenic bacteria were enriched in *both* CDI and non-CDI patients. A previously unreported finding of increased numbers of *Akkermansia muciniphila* in CDI patients was observed, an organism which degrades mucin and which therefore may provide a selective advantage toward CDI. Fungal elements of the genus *Penicillium* were predominant in CDI; these organisms produce antibacterial chemicals which may resist recovery of healthy microbiota. The most frequent CDI microbial community networks involved *Peptostreptococcaceae* and *Enterococcus*, with decreased population density of *Bacteroides*. These results suggest that the development of CDI is associated with microbiota changes which are consistently associated with CDI in human subjects. These gut taxa contribute to the intestinal dysbiosis associated with *C. difficile* infection.

## Introduction

The community of indigenous microorganisms (including bacteria, viruses, and fungal elements) which co-exist within a host’s gastrointestinal tract is termed the *gut microbiome*. Disturbances to the gut microbiota, particularly by antibiotics, have been linked to the development of CDI (Peterfreund et al., 2012; Theriot et al., 2014). In healthy patients, normal indigenous microbes have been hypothesized to prevent disease states through alleged mechanisms such as direct pathogen inhibition, depletion of pathogen required nutrients, and stimulation of the host immune system (Stecher and Hardt, 2011). However, the majority of studies evaluating changes to these gut microbial communities in the setting of CDI have either been limited to niche human populations, such as infants or the elderly, or they have resorted to murine models (Reeves et al., 2011; Rea et al., 2012; Rousseau et al., 2012; Lawley and Young, 2013; Koenigsknecht et al., 2015; Miezeiewski et al., 2015). From this relatively small body of published literature, there is a consensus that a common feature distinguishing the CDI patient from a healthy subject is a significant decrease in both population diversity and density of bacterial species characterized by a loss of frequently encountered commensal bacteria and an overabundance of certain anaerobes (*Proteobacteria*; Skraban et al., 2013; Zhang et al., 2015).

There are four elements to current microbiome studies which limit their clinical relevance. First, a significantly under-represented feature of most published microbiome studies relates to an analysis of the altruistic or adversarial relationships between bacterial community structures in the gut of the same patient. The compositional profile of individual and aggregate communities within the gut can shift the environment of the large intestine toward a more or a less hospitable habitat for pathogenic bacteria such as *C. difficile* through a number of different mechanisms (such as availability of carbon and food sources, effects on mucin and bacterial toxin production; Perez-Cobas et al., 2014). A second factor which is often unaccounted for in previous studies on CDI relates to the role of fungal elements. Fungal species are a significant proportion of the gut microbial environment but their involvement in the development of CDI is virtually unstudied. Thirdly, there are few studies on CDI and the microbiome which use stool from human subjects, due to the challenges of collecting those tissue samples and accruing cohorts with an acceptable degree of homogeneity which would afford proper comparison. Fourthly, studies of the microbiome in CDI require *C. difficile* negative *diarrheal* stools as a proper control. This feature is important since diarrhea, with or without *C. difficile*, is associated with changes to the microbiota which are not present in patients with formed stool; additionally, it is patients with diarrhea who will be undergoing testing for CDI, and ensuring both cohorts have similar symptoms is important for veridical comparisons.

The current study hypothesizes that CDI as a disease state of the gut is associated with certain bacterial and fungal community structures which are consistent between human hosts. The aim of this study was to compare and contrast different microbial taxa and various microbial network co-occurrence patterns observed among prospectively collected, human derived *C. difficile* positive and negative diarrheal stool samples. It is postulated that this information would prove valuable in better understanding the mechanism of how previously reported changes in gut taxa lead to CDI, as well as potentially providing targets for prevention and treatment.

## Materials and Methods

### Study Design and Specimen Collection

This prospective cohort study was conducted exclusively at the Penn State Hershey Medical Center with approval from the Institutional Review Board. Diarrheal stool samples collected from hospitalized medical and surgical patients were submitted to the Hershey Medical Center Microbiology laboratory for *C. difficile* testing between June 2014 and November 2014 and were considered for inclusion in this study. Each patient signed an IRB approved consent form prior to their stool being collected for use in this study. Within 12 h of stool collection, specimens were screened for *C. difficile* using a NAAT designed to detect a highly conserved region of the *C. difficile tcdA*. Detection of *tcdA* was considered positive for CDI, while absence of detection of *tcdA* was considered to be negative for CDI, mirroring clinical practice in the senior author’s hospital. A positive or a negative NAAT was the basis for forming the CDI and control groups. A colleague in the clinical microbiology lab of our institution who oversaw all testing of inpatient stool samples for CDI would contact members of our team on a daily basis regarding positive and negative samples. These samples awaiting retrieval in the clinical microbiology lab would be preserved in a -80°C freezer. A member of our team would, as soon as possible, then attempt to consent that patient for permission to use their stool sample in this study, as well as receiving consent to access their electronic medical record. Once consent was obtained, the stool sample was moved directly from a -80°C freezer in the clinical microbiology lab to a -80°C freezer in the study senior author’s research lab. These samples were stored until all study samples were collected, which occurred in November 2014, using this approach so that the samples could be thawed, and processed for sequencing during one period. Twelve patients comprised each cohort; additionally, one patient provided a *C. difficile* positive stool sample, and after successful treatment with a confirmatory negative NAAT test, this subject’s stool was collected again after CDI therapy for post-treatment analysis. In all, there were 13 samples in each of the two cohorts.

Inclusion criteria for both cohorts included patients 18 years of age or older who submitted a diarrheal stool sample for CDI testing as part of each patient’s routine clinical care. Exclusion criteria included an age younger than 18 years, a gravid status, the inability to provide informed consent, or the administration of *difficile*-directed antibiotics prior to a stool sample being collected. The research team was notified of candidate samples on a daily basis, allowing for informed consent from potential study subjects to be obtained in a timely fashion. Following the consent process, stool samples were immediately obtained from the Microbiology laboratory and were stored in the laboratory of the principal investigator (DBS) at -70°C for use in the present study.

### PCR Amplification

DNA was extracted from approximately 0.25 g of fecal material using the MoBio Powerfecal^TM^ DNA Extraction Kit following the manufacturer’s instructions (MoBio, Carlsbad, PA, USA). Illumina iTag PCR reactions (25 μL) contained ∼5–10 ng of template DNA and a final concentration of 1x PCR buffer, 0.8 mM dNTP’s, 0.625 U Taq polymerase, 0.2 μM 515F forward primer and 0.2 μM illumina 806R reverse barcoded primer, per reaction. PCR was carried out on an MJ Research PTC-200 thermocycler (Bio-Rad, Hercules, CA, USA). Pooled PCR products were gel purified using a Qiagen Gel Purification Kit (Qiagen, Frederick, MD, USA) and were quantified using a Qubit 2.0 fluorometer (Life Technologies, Carlsbad, CA, USA). Prior to submission for sequencing, libraries were quality checked using the 2100 Bioanalyzer DNA 1000 chip (Agilent Technologies, Santa Clara, CA, USA).

### Sequencing

Library pools were size verified using the Fragment Analyzer CE (Advanced Analytical Technologies Inc., Ames IA, USA) and were quantified using a Qubit high sensitivity dsDNA kit (Life Technologies, Carlsbad, CA, USA). After dilution to a final concentration of 1 nM containing 10% PhiX V3 library control (Illumina, San Diego, CA, USA), the library pools were denatured for 5 min in an equal volume of 0.1N NaOH, were further diluted to 12 pM in HT1 buffer (Illumina) and were sequenced using an Illumina MiSeq V2 300 cycle kit cassette with 16S rRNA library sequencing primers set for 150 base, paired-end reads.

### Quality Filtering/OTU Picking

Sequence reads were trimmed at a length of 150 base pairs and were quality filtered at an expected error of less than 0.5% using USEARCH v7 (Edgar, 2013). After quality filtering, reads were analyzed using QIIME 1.9.0 (Caporaso et al., 2010, 2011). Chimeric sequences were identified using USEARCH61 (Edgar, 2010). A total of 1,372,573 sequences were retrieved after quality filtering and chimera checking. Open reference OTUs were picked using the USEARCH7 algorithm (Edgar, 2010), and taxonomy assignment was performed using the Greengenes 16S rRNA gene database (13-5 release, 97%; DeSantis et al., 2006). Clustered taxa were compiled into an OTU table. The unrarified OTU table underwent CSS normalization for beta diversity analysis.

### ITS Library Preparation and Bioinformatics Analysis for Fungal Elements

ITS fungal community analysis was performed using the ITS1-F and barcoded ITS2 primers as described in Bellemain et al. (2010). Illumina iTag (PCR were performed at a total volume of 25 μL for each sample and contained final concentrations of 1X PCR buffer, 0.8 mM dNTPs, 0.625 U Taq, 0.2 μM ITS1-F forward primer, 0.2 μM reverse barcoded ITS2 primer and ∼10 ng of template DNA per reaction. PCR was carried out on a MJ Research PTC-200 thermocycler, and PCR products were pooled and purified as described for 16S rRNA gene libraries. OTUs were picked *de novo* using the UPARSE algorithm with singleton reads discarded as recommended by Edgar (2013). The OTUs in the data set were compared against the fungal ITS UNITE database using the BLAST.

### Microbial Community Analysis

Principal coordinates analysis plots and ANOSIM tests for significance were calculated using a weighted UniFrac distance matrix generated from a CSS normalized OTU table within Qiime 1.9.0. The non-parametric Kruskal–Wallis test for significance was used to identify enriched taxa within control and CDI patients. Differences were considered to be significant at an alpha of 0.01. Relative abundance plots were produced from a CSS normalized OTU table (Paulson et al., 2013). LEfSe was also used to identify taxonomic biomarkers between control and CDI patients (Segata et al., 2011). Genus-level relative abundances were multiplied by one million and were formatted as described in Segata et al. (2011). Comparisons were made with “Status” (control or CDI) as the main categorical variable. Alpha levels of 0.10 were used for the Kruskal–Wallis tests, and Linear Discriminant Analysis scores for the enriched taxa within each class were plotted. Features were plotted on a logarithmic scale according to the experimental group to which they were significantly associated. LEfse explicitly requires all pairwise comparisons to reject the null hypothesis for detecting the biomarker taxa; thus, no multiple testing corrections were needed (Segata et al., 2011). Alpha diversity indices, including Chao1, Heip’s evenness, Observed Species, and PD Whole Tree, were generated from an unrarified OTU table with a max sampling depth of 1900, a step size of 190, and with 25 iterations at each step. Core microbiome analyses were conducted on an unrarified OTU table within qiime-1.9.0. Network plots were generated within Cytoscape 3.2.0 from a filtered OTU table rarified at a sequencing depth of 3218 within Qiime1.9.0. Only OTUs identified at the species level were kept for network analysis, all remaining taxa were filtered within Qiime. The Cytoscape style Biopax was utilized for data visualization.

A co-occurrence network was created within the Cytoscape plugin Conet from an unrarified OTU table containing bacterial abundance data from twelve CDI patients (Faust et al., 2012). All taxa unassigned at the kingdom taxonomic ranking were discarded. Spearman’s correlations between taxa abundances were calculated, and only strong correlations (Spearman’s rho > ±0.8) were included in the network. Minimum OTU occurrence was set to 10, discarding all OTUs not observed 10 times within each sample.

### Sample Size Calculation

While there is a relative dearth of previous publications which could guide a sample size estimate for a study focused on inpatients with diarrhea, assuming a richness of 1,000 OTUs at a power of 0.8, 10 subjects per cohort would require a minimum change in abundance of 2.5-fold for significance. The present study surpasses this requirement.

## Results

### Description of Study Population

The stool samples of twelve patients with CDI and twelve patients without CDI were used in this study. **Table 1** outlines the clinical characteristics of these patients. All patients enrolled in this study had diarrheal stools, and the two cohorts demonstrated no significant differences in terms of the selected clinical characteristics. There was no disparity in any risk factor for CDI, or for other causes of diarrhea, identified between the cohorts.

**Table 1:** Patient characteristics.

Variable	CDI (*n* = 12)	Diarrheal control (*n* = 12)	*P*-value
Age, mean [SD]	55.5 ± 20.5	51.2 ± 16.6	NS
Sex			NS
Female	5 (41.7)	7 (58.3)	
Male	7 (58.3)	5 (41.7)	
Body mass index (kg/m^2^), mean [SD]	29.3 ± 7.8	34.3 ± 13.3	NS
Hospitalized within last 3 months	8 (66.7)	7 (58.3)	NS
Surgery within last 6 months	3 (25)	3 (25)	NS
Antibiotic use within last 3 months	6 (50)	7 (58.3)	NS
Amoxicillin/clavulanate	1 (8.3)	0	
Cephalosporin	4 (33.3)	3 (25)	
Fluoroquinolone	2 (16.7)	2 (16.7)	
Intravenous vancomycin	1 (8.3)	3 (25)	
Trimethoprim– sulfamethoxazole	0	3 (25)	
Proton pump inhibitor use within last 3 months	9 (75)	8 (66.7)	NS
History of *Clostridium difficile* infection	3 (25)	0	NS
History of inflammatory bowel disease	1 (8.3)	1 (8.3)	NS
Length of hospital stay prior to specimen collection (days), mean [SD]	5 ± 4	3 ± 2	NS


*Values within parentheses represent percentages unless indicated otherwise.*

### Assessment of Alpha and Beta Diversity of Bacterial Microbiota

**FIGURE 1:**
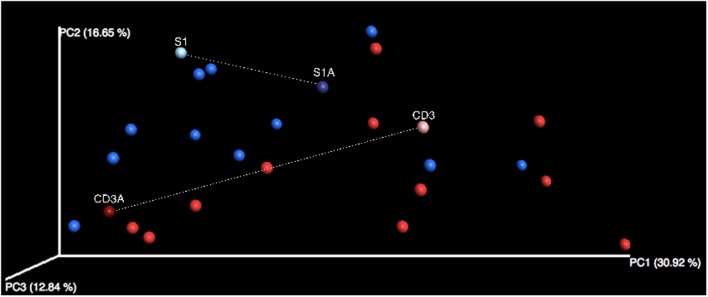
**Principal Coordinates Analysis in non-CDI and CDI fecal samples.** Weighted UniFrac distance matrices were generated from a filtered OTU table that underwent CSS normalization. Control samples (*n* = 13) are displayed in blue, while CDI samples are in red (*n* = 13). Distinct clustering can be observed between bacterial communities of these two groups. Sample S1 (no antibiotics) and S1A (antibiotics) are fecal samples from the same control individual. Sample CD3 (no antibiotics) and CD3A (antibiotics) are fecal samples from the same individual with CDI.

When comparing observed richness and evenness in control subjects versus CDI patients, two-sample non-parametric *t*-tests demonstrated no significant difference between the two groups (richness: *P* = 0.224; evenness: *P* = 0.760). Beta diversity, however, did reveal differences in the bacterial community structure between the control and CDI cohorts as demonstrated by the distinct clustering observed between the two cohorts on PCoA (**Figure 1**). Analysis of similarities confirmed that this clustering was significant (*P* = 0.004), though these differences in bacterial community structures did not vary significantly based on either age (*P* = 0.644) or sex (*P* = 0.603).

**FIGURE 2:**
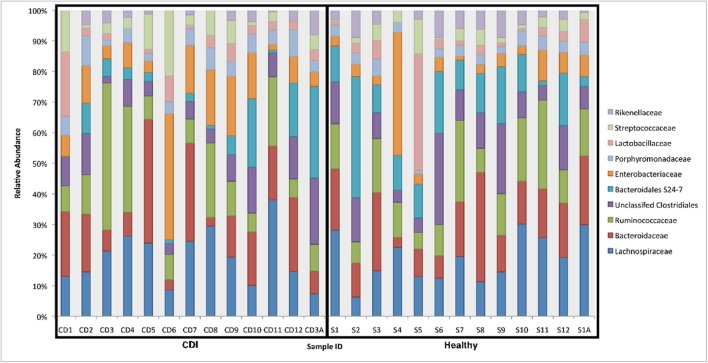
**Relative abundance of bacterial families in CDI and non-CDI individuals.** The relative abundance of the top 10 most abundant bacterial families for CDI (*n* = 13) and control (*n* = 13) fecal samples. Relative abundance of taxa was determined based on the relative abundance of 16S rRNA gene sequences assigned to a given bacterial family using the Greengenes database.

Fecal samples from both cohorts were dominated by *Lachnospiraceae*, *Bacteroidaceae*, and *Ruminococcaceae* families (**Figure 2**). Non-parametric Kruskal–Wallis tests identified specific taxa that were enriched in either of the cohorts. Interestingly, three different OTUs from the *Peptostreptococcaceae* matching closely (>97%) to *C. difficile* strain 630 were significantly enriched (*P* < 0.005) in CDI fecal samples. In addition, two OTUs, *Akkermansia muciniphila* and an unknown *Enterobacteriaceae*, were also more abundant in CDI individuals. A plethora of taxa were significantly enriched in the control cohort, including members of the *Bacteroidales* and *Clostridales* groups.

**FIGURE 3:**
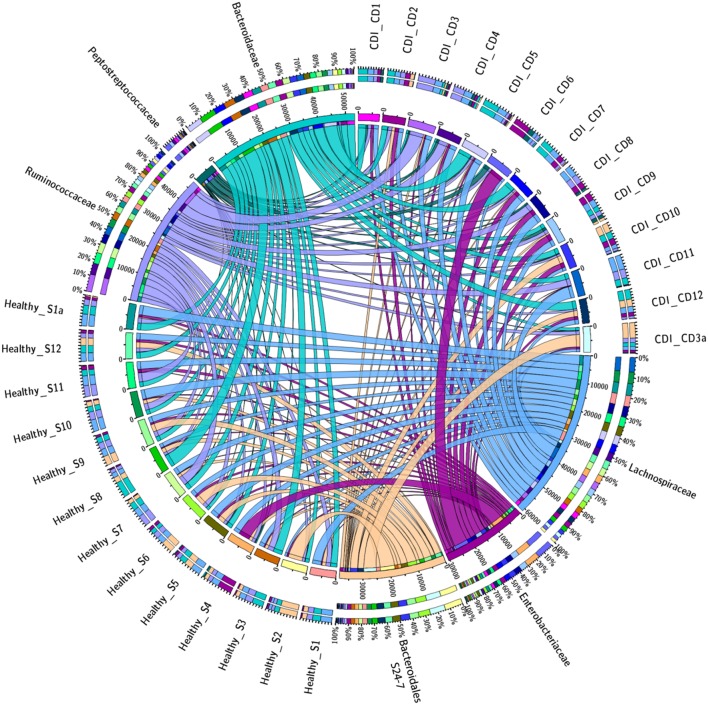
**Prevalence of specific bacterial families in CDI and non-CDI cohorts.** The circos plot displays relative abundance of bacterial orders within control and CDI samples. Plots were generated from a CSS normalized OTU table with all singletons removed. The abundance of each order is directly proportional to the width of each ribbon connecting bacterial taxa to its respective sample. Each bacterial family is assigned a specific color. The outer ring represents the cumulative percent of 16S sequences assigned to a given family from each sample, while the inner circle represent the number of 16S rRNA sequences assigned to a given taxa in a given sample.

**FIGURE 4:**
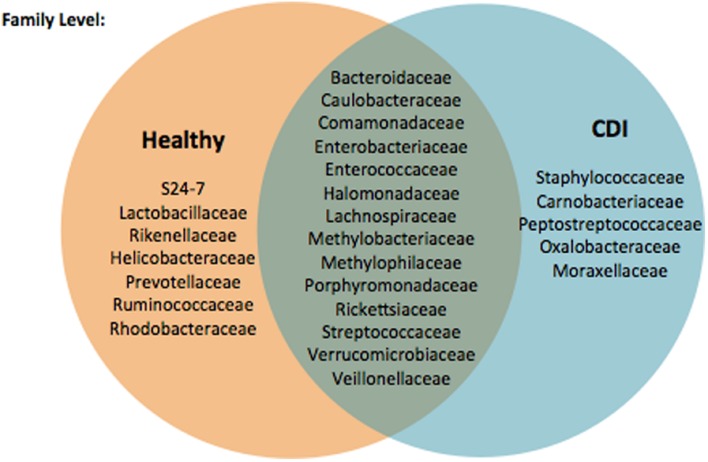
**Venn diagram demonstrating distinct and shared OTUs between non-CDI and CDI states**.

Fewer OTUs were shared among CDI individuals as compared to control patients, suggesting several members of the core microbiome are lacking in CDI, and further analysis of the prevalence and abundance of the specific bacterial families within the study cohorts demonstrated that *Enterobacteriaceae* and *Peptostreptococcaceae* were more abundant in CDI individuals as compared to non-CDI individuals (**Figure 3**). In contrast, *Bacteroidales* family S24-7 was enriched in non-CDI individuals. **Figure 4** provides a Venn diagram breakdown demonstrating the distinct and shared OTUs between CDI and non-CDI states.

### Fungal Community Results

Of the nine OTUs found to be significantly enriched in the infected cohort, eight unique OTUs were matched to unknown fungal taxa labeled as “uncultured fungus,” with these data suggesting that a defined fungal community of currently unknown phylogenetic origin comprised a significant portion of the fungal community within patients affected with CDI. The only enriched taxa within the CDI cohort that was successfully mapped to the fungal database belonged to the *Penicillium* genus. In summary, a small number of commonly detected species were detected in non-CDI subjects, while a larger number of rare taxa were pervasive in the CDI group.

### Community Structure and Co-Occurrence Analysis

**FIGURE 5:**
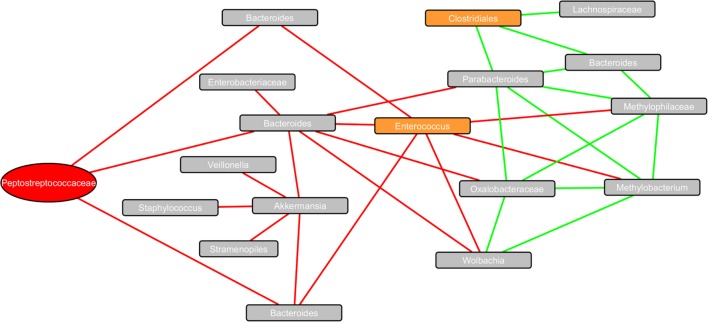
**Co-occurrence network plot of bacterial taxa within the fecal microbiome of CDI patients.** The co-occurrence network plot generated within the Cytoscape plugin *Conet* reveals strong positive (Spearman’s rho > 0.8) and strong negative (Spearman’s rho < -0.8) correlations between OTUs identified within 13 CDI stool samples. Eighteen unique OTUs (indicated by rectangular nodes) possessing strong correlations with one another are shown. The 16S rRNA sequence of the *Peptostreptococcaceae* node highlighted in red matched *Clostridium difficile* strain 630 (query cover = 100%) when BLAST searched. Additional potential pathogenic OTUs are shown in orange, including *Enterococcus* and an unclassified *Clostridiales*. Edges connecting nodes highlighted in green are indicative of a strong positive correlation, whereas edges highlighted in red are indicative of a strong negative correlation.

The co-occurrence network (**Figure 5**) revealed strong correlations between bacterial taxa (communities) within the fecal microbiome of CDI patients. Taxa of interest within the infected cohort included unclassified *Peptostreptococcaceae*, which matched *C. difficile* strain 630 when BLAST searched (100% query)*, Enterococcus* and unclassified *Clostridiales*. Interactions with *Peptostreptococcaceae* revealed strong negative correlations with three different *Bacteroides* OTUs, including an OTU matching to *Bacteroides fragilis*. *Enterococcus* was also negatively correlated to the same *Bacteroides* strains, as well as *Wolbachia, Methylophilaceae*, and *Methylobacterium adhaesivum*. *Clostridiales* OTUs had strong positive correlations with *Parabacteroides, Bacteroides*, and *Lachnospiraceae*.

### Antibiotic Effect on Community Structure

A single patient from the CDI cohort underwent successful treatment with 10 days of antibiotics (metronidazole). A second stool sample was collected 12 days after the conclusion of CDI antibiotic therapy and was confirmed to be negative for *C. difficile* based on nucleic acid amplification testing for *tcdA*. The microbial community following successful treatment for CDI demonstrated a shift toward the same microbial community structures common among the non-CDI cohort. The greatest shifts observed were a fourfold increase in unclassified *Acetobacteraceae* in response to antibiotic intervention, and a fourfold decrease in *Myxococcales OM27* in response to treatment.

A total of 6 of the 11 most abundant genera within this treated subject observed a twofold shift in response to antibiotic treatment. The most dramatic increases (>10%) within this group of 11 taxa were observed within the *Bacteroidales* S24-7 and *Clostridiales*. The *Ruminococcaceae* and *Lachnospiraceae* presented the greatest decrease (>7%) in response to antibiotics.

## Discussion

The results of this study can be summarized as follows. In contradistinction (Antharam et al., 2013) to several previous publications, our patient population was found to have a relatively rich abundance of butyrogenic bacteria (such as *Lachnospiraceae* and *Ruminococcaceae*) in *both* the CDI and non-CDI cohorts, though the non-CDI cohort was more replete with the organisms. CDI patients were noted to have a predominance of anaerobes of the *Peptostreptococcaceae* family, with a relative depletion of anaerobes of the *Bacteroidales* and *Clostridiales* groups. A previously unreported finding of increased numbers of *Akkermansia muciniphila* in CDI patients was observed, as was an increase in several unknown species of *Enterobacteriaceae*. Fungal OTUs have not been previously reported in the CDI population, and this study identified fungal elements of the genus *Penicillium* as a predominant feature of CDI. Within CDI patients, the microbial community networks most frequently encountered were *Peptostreptococcaceae* and *Enterococcus*, to the exclusion of *Bacteroides*. Lastly, following successful treatment of a CDI patient, comparison of microbial communities before and after treatment reveal a shift toward greater numbers of *Bacteroides* and butyrogenic bacteria, similar to the microbial communities encountered in non-CDI patients, providing a causal description for the microbiota changes associated with successful antibiotic treatment for CDI.

There are several interesting observations from this study. One important reflection from a review of **Table 1** reveals an equal distribution of frequently cited clinical risk factors for CDI (Thibault et al., 1991; Bignardi, 1998; Hegarty et al., 2014; Regnault et al., 2014) between both the infected and uninfected cohorts. In this study of inpatients with diarrhea, the use of, and type of antibiotic, use of proton pump inhibitors, previous diagnosis of CDI, diagnosis of inflammatory bowel disease and length of stay did not significantly differ between CDI and non-CDI patients with diarrhea. While these clinical variables are associated risk factors for CDI, their discriminatory power for inpatient populations may be limited given the large number of diarrheal patients with and without CDI who share these factors. This observation may indicate that diarrhea among inpatients should probably reflex to CDI testing, as any attempt at stratifying a patient’s risk for CDI based on clinical factors lacks specificity to reliably dispense with stool testing.

There have been virtually no studies investigating shifts in fungal microbiota in diarrheal patients, with or without CDI. The present study demonstrated that in CDI patients, OTUs associated with the genus *Penicillium* were prominent. This fungus is associated with diarrhea in immunocompromised patients (Leung et al., 1996). Studies assessing the effect of antibiotics on the hamster gut microbiome, such as in the setting of clindamycin used to induce CDI, have reported that *Penicillium* was a predominant fungal element (Peterfreund et al., 2012). The present study is the first to describe this same association in human subjects, and the *Penicillium* genus may be a potential biomarker for CDI infection in humans with diarrheal illnesses. Interestingly, *Penicillium* is useful in environmental microbiology due to its utility in bioremediation, as it produces enzymes which degrade a number of xenobiotic compounds, all of which can prevent bacterial colonization and survival (Leitao, 2009). This effect of *Penicillium* on ambient bacteria could represent a causal mechanism by which this fungus may, in conjunction with disturbances to the normal microbiota, help to further promote intestinal dysbiosis as well as resisting shifts back toward healthy gut ecologies.

The predominance of *Akkermansia muciniphila* in CDI patients is a novel observation in human subjects. Murine models have noted that changes to the normal thickness and constitution of colonic mucus, especially within the right colon, have effects on colonization of the large intestine with normal microbiota (Semenyuk et al., 2015). A normal mucus layer is essential to the establishment and preservation of non-pathogenic gut associated communities commensurate with normal health. *A. muciniphila* is a well-known mucin-degrader, which may provide a selective advantage to *C. difficile* in establishing a predominant population within the gut (Derrien et al., 2004, 2008; van Passel et al., 2011). It has recently been reported that *C. difficile* can itself reduce the production of mucin, while also altering the composition of mucus to allow additional *C. difficile* an advantage in adhering to the altered mucus as opposed to other beneficial organisms (Engevik et al., 2015). The co-occurrence of *A. muciniphila* in CDI patients may be a result of initial shifts toward the CDI disease state, with an increase in pathogenic bacteria such as *A. muciniphila* leading to the entrenchment of pathogenic gut communities via changes to colonic mucus. This may be one of several potential reasons that *C. difficile* infection is so resistant to treatment, due to the co-emergence of other bacteria that are not susceptible to *difficile*-directed antibiotics and that promote a gut environment with barriers to reverting to a healthy ecology.

The strong negative correlation between *C. difficile* and *Bacteroides* in this study is of interest, as the *Bacteroides* are abundant and mutualistic members of the human gut microbiome, being involved in key metabolic processes involved in normal intestinal health, including carbohydrate fermentation and polysaccharide production (Wexler, 2007). One reason *Bacteroides* excel at dominating the gut microbiota relates to their ability to modulate surface polysaccharides in an effort at evading host immune systems (Wexler, 2007). Furthermore, *B. fragilis* has been shown to promote growth of gut-associated lymphoid tissue by producing polysaccharides that can activate CD4^+^ T cells which assist in directing lymphocytes to sites of infection (Round and Mazmanian, 2009). Rousseau et al. (2011) observed a similar trend in a cohort of infants, where a low *Firmicutes* to *Bacteroides* ratio and an increase in facultative anaerobes was associated with an observed increase in colonization by *C. difficile*. In this study, a strong positive correlation with unclassified *Clostridiales* was found. Our data suggest that *C. difficile* may actually repress the growth of commensal bacteria such as *Bacteroides* based on community modeling in the CDI cohort.

Analysis of the microbiota community of a CDI positive individual following 10 days of *difficile*-directed antibiotic therapy with metronidazole revealed the selective enrichment of *Bacteroidales* S24-7 within the post-antibiotic stool sample. Interestingly, this order of bacteria are known to produce butyrate, a short chain fatty acid that has been shown to promote colonic barrier strength at appropriate concentrations by increasing mucin production, decreasing colonic permeability, and thereby reducing the susceptibility of the colon to infections (Hatayama et al., 2007). In prior studies, the relative depletion of butyrogenic bacteria has been described to play a key role in the conversion of the intestinal microenvironment to one that favors the germination and growth of *C. difficile* (Antharam et al., 2013; Zhang et al., 2015; Gu et al., 2016). The predominance of *Bacteroidales* may be a biomarker signaling a shift in the gut environment that coincides with eradication of *C. difficile* infection, and the effect of butyrate on promoting gut health may be one of the mechanisms for this effect. It should be noted that our data on microbiota changes in CDI patients after antibiotic therapy is very limited by the current study analyzing only one sample from one patient following successful antibiotic therapy for CDI.

The present study is limited by being performed at a single medical center. Additionally, although our number of subjects surpasses the minimum necessary based on our sample size calculations, the present study included a smaller number of subjects due to the expense of a study of this type. Since the current study population was limited to inpatients, many of the study subjects were placed on antibiotics for various reasons, and the use of antibiotics can significantly affect the composition of gut microbiota. The current study population, as described in **Table 1**, harbored no statistically significant differences in any of the indices listed in this table, including the incidence of antibiotic use as well as the type of antibiotic used. In an effort to have homogenous cohorts, we limited our study to those with diarrhea, and as **Table 1** demonstrates, these groups of patients were quite similar in terms of demographics, chronic health conditions and CDI risk factors. Secondly, this is not the first study to describe changes in OTUs based on 16S rRNA sequencing. Our study attempted to go beyond this by including information on fungal elements, and by focusing on how communities of bacterial and fungal microbiota differ between CDI and non-CDI diarrheal states. This, coupled with our focus on inpatients with diarrhea, provide not only novelty, but a study that speaks to the patients who most frequently undergo CDI testing.

## Conclusion

*Clostridium difficile* infection is associated with reproducible and consistent changes in the microbiome, including the rise of pathogenic anaerobic organisms (*Peptostreptococcaceae* and *Enterococcus*), mucin degrading bacteria (*A. muciniphila*) and a predominant fungal element (*Penicillium*). These microbes may exert a synergistic effect, providing *C. difficile* with a selective advantage by creating multiple barriers toward reversal of the intestinal dysbiosis associated with the CDI disease state. These gut taxa contribute to the intestinal dysbiosis associated with *C. difficile* infection.

## Author Contributions

Guarantor of the article: DS Study concept and design: WS, RL, JH, and DS. Acquisition and analysis of data: WS, JH, RL, and DS. Interpretation of data: all authors. Drafting of the manuscript: WS, RL, and DS. Critical revision of the manuscript for important intellectual content: all authors. Study supervision: RL and DS.

## Conflict of Interest Statement

The authors declare that the research was conducted in the absence of any commercial or financial relationships that could be construed as a potential conflict of interest.

## Abbreviations

ANOSIM: analysis of similarity
BLAST: basic local alignment search tool
CDI: *Clostridium difficile* infection
CSS: cumulative sum scaling
LEfSe: Linear Discriminant Analysis effect size
NAAT: nucleic acid amplification test
OTUs: operational taxonomic units
PCoA: principal coordinates analysis
PCR: polymerase chain reactions
*tcdA*: gene for toxin A
